# Anxiety and depression in patients with gastrointestinal cancer: does knowledge of cancer diagnosis matter?

**DOI:** 10.1186/1471-230X-7-28

**Published:** 2007-07-14

**Authors:** Azadeh Tavoli, Mohammad Ali Mohagheghi, Ali Montazeri, Rasool Roshan, Zahra Tavoli, Sepideh Omidvari

**Affiliations:** 1 Department of Psychology, Faculty of Humanity Studies, Shahed University, Tehran, Iran; 2 Cancer Research Centre, Cancer Institute, Tehran, Iran; 3 Iranian Institute for Health Sciences Research, Tehran, Iran

## Abstract

**Background:**

Gastrointestinal cancer is the first leading cause of cancer related deaths in men and the second among women in Iran. An investigation was carried out to examine anxiety and depression in this group of patients and to investigate whether the knowledge of cancer diagnosis affect their psychological distress.

**Methods:**

This was a cross sectional study of anxiety and depression in patients with gastrointestinal cancer attending to the Tehran Cancer Institute. Anxiety and depression was measured using the Hospital Anxiety and Depression Scale (HADS). This is a widely used valid questionnaire to measure psychological distress in cancer patients. Demographic and clinical data also were collected to examine anxiety and depression in sub-group of patients especially in those who knew their cancer diagnosis and those who did not.

**Results:**

In all 142 patients were studied. The mean age of patients was 54.1 (SD = 14.8), 56% were male, 52% did not know their cancer diagnosis, and their diagnosis was related to esophagus (29%), stomach (30%), small intestine (3%), colon (22%) and rectum (16%). The mean anxiety score was 7.6 (SD = 4.5) and for the depression this was 8.4 (SD = 3.8). Overall 47.2% and 57% of patients scored high on both anxiety and depression. There were no significant differences between gender, educational level, marital status, cancer site and anxiety and depression scores whereas those who knew their diagnosis showed a significant higher degree of psychological distress [mean (SD) anxiety score: knew diagnosis 9.1 (4.2) vs. 6.3 (4.4) did not know diagnosis, P < 0.001; mean (SD) depression score: knew diagnosis 9.1 (4.1) vs. 7.9 (3.6) did not know diagnosis, P = 0.05]. Performing logistic regression analysis while controlling for demographic and clinical variables studied the results indicated that those who knew their cancer diagnosis showed a significant higher risk of anxiety [OR: 2.7, 95% CI: 1.1–6.8] and depression [OR: 2.8, 95% CI: 1.1–7.2].

**Conclusion:**

Psychological distress was higher in those who knew their cancer diagnosis. It seems that the cultural issues and the way we provide information for cancer patients play important role in their improved or decreased psychological well-being.

## Background

Cancer is well known to be a difficult disease, affecting patients and their families both physically and emotionally. Despite biomedical progress, cancer is still often considered synonymous with death, pain and suffering [1]. It is argued that cancer is not just a single event with a certain end but a permanent condition characterized by ongoing ambiguity, potentially delayed or late effects of the disease or its treatment and concurrent psychological issues [2]. Previous studies have demonstrated an increased risk for psychiatric morbidity among cancer patients [3,4]. The prevalence of psychiatric disorders in cancer patients varies greatly among studies ranging from 9% to 60% [3,5,6], although in large studies using standardized psychiatric interviews and applying research diagnostic criteria the range narrows from 10% to 30% [7].

There are few studies that examined anxiety and depression in gastrointestinal cancer patients. Nordin et al. in their studies of gastrointestinal cancer patients have shown that overall 17% of gastrointestinal patients suffer from anxiety and 21% experience depression and that those with gastric cancer are more vulnerable to psychological distress in connection with the diagnosis than are colorectal cancer patients [8,9]. They also showed that there are only minor changes over time in the average values of emotional well being in these patients [10]. In addition they have indicated that levels of anxiety and depression at diagnosis could predict a similar status 6 months later [11]. Importantly they found that patients' satisfaction with life, as defined in terms of the discrepancy between the perceived attainment and subjective importance of various life values, is associated with anxiety and depression [12]. However, recent studies have reported that anxiety is more common in younger patients and depression in those who experience long-term hospitalization and that coping style might be an important factor in contributing to the level of anxiety and depression in gastrointestinal cancer patients [13,14].

The incidence of esophageal and stomach cancer in Iran is high, well above the world average [15]. It is the first leading cause of cancer related deaths in men and the second among women [16]. This paper reports the results of a study that aimed to investigate about psychological state of Iranian gastrointestinal cancer patients; and to examine the relationship between various demographic and clinical factors and psychological distress in this group of patients. In particular this study aimed to investigate the role of knowledge about the cancer diagnosis and to compare anxiety and depression in patients who knew their cancer diagnosis and those who did not.

## Methods

### Design and data collection

An interview based prospective study was carried out to measure anxiety and depression in patients with gastrointestinal cancer. Data were collected during November 2005 and April 2006. The intention was to interview all gastrointestinal cancer inpatients attending a large teaching hospital (Imam Hospital) in Tehran, Iran. A psychologist in a face-to-face interview administered the questionnaire. Data on demographic characteristics and clinical information including age, gender, educational status, cancer site and time since diagnosis were extracted from case records. To assess patients' knowledge of the cancer diagnosis, both patients and relatives were investigated separately. This was achieved at the end of each interview. First we asked relatives to indicate whether a patient knew his or her diagnosis. Then to confirm this with patients, after a careful consideration each patient was asked what was wrong with he or she. Knowledge was assessed by patients' ability to acknowledge the illness and use the terms "cancer "or "tumor".

All participants in the study were gastrointestinal cancer patients who were diagnosed during one year ago. Patients who had cognitive problems or were too sick to participate in the interview were excluded. Verbal consents obtained from all patients prior to interview. The Ethics Committee of the Tehran University of Medical Sciences approved the study.

### Instruments

Anxiety and depression was measured using the Hospital Anxiety and Depression Scale (HADS) [17]. This is a widely used valid questionnaire to measure psychological distress in cancer patients [18]. Also it is a brief and well-established instrument with cut-off suggestive of a psychiatric diagnosis [19]. The validation study of the Iranian version of the HADS proved that it is an acceptable, reliable and valid measure of psychological distress [20]. The HADS is a 14-item questionnaire consisting of two subscales: anxiety and depression. Each item is rated on a four-point scale giving maximum scores of 21 for anxiety and depression. Scores of 11 or more on either subscale are considered to be a significant "case" of psychological morbidity, while scores of 8–10 represents "borderline", and 0–7 "normal". We administered the HADS in an interview format.

### Statistical analysis

The one-way analysis of variance (ANOVA with Bonferroni correction), and independent samples t-test were performed to compare anxiety and depression with regard to gastrointestinal cancer patients' demographic and clinical characteristics. In addition logistic regression analysis was carried out  in order to examine which factors show the strongest association with anxiety and depression. For the purpose of the analysis relative to the recommended cut-off points patients were divided into two groups: those who scored 0 to 7 as normal and those who scored 8 and above as probable case [21,22]. Data were analyzed using the SPSS software version 13.0.

## Results

### Patients' characteristics

In total 142 patients were studied. The mean age of patients was 54.1 (SD = 14.8) years, most patients were married (86%), male (56%), illiterate (55%); and 48% knew their cancer diagnosis, whereas 52% did not know. The diagnosis were as fallows: stomach (30%), esophagus (29%), colon (22%), rectum (16%), and small intestine (3%). The patients' demographic and clinical characteristics are shown in Table 1.

**Table 1:** Gastrointestinal cancer patients' demographic and clinical characteristics

	**Knew diagnosis (n = 68)**	**Did not know diagnosis (n = 74)**	**All (n = 142)**
	**No. (%)**	**No. (%)**	**No. (%)**

**Age**			
Mean (SD)	50.2 (13.9)	58.2 (13.4)	54.1 (14.8)
Range	23–74	19–76	19–76
**Gender**			
Male	34 (50.0)	45 (60.8)	79 (55.6)
Female	34 (50.0)	29 (39.2)	63 (44.4)
**Marital status**			
Single	7 (10.3)	6 (8.1)	13 (9.2)
Married	59 (86.8)	63 (85.1)	122 (85.9)
Widowed	2 (2.9)	5 (6.8)	7 (4.9)
**Educational status**			
Illiterate	23 (33.8)	55 (74.2)	78 (54.9)
Primary	28 (41.2)	15 (20.3)	43 (30.3)
Secondary	9 (13.2)	3 (4.1)	12 (8.5)
College/university	8 (11.8)	1 (1.4)	9 (6.3)
**Cancer site**			
Esophagus	7 (10.3)	34 (45.9)	41 (28.9)
Stomach	22 (32.4)	20 (27.0)	42 (29.6)
Small intestine	1 (1.5)	4 (5.4)	5 (3.5)
Colon	26 (38.2)	5 (6.8)	31 (21.8)
Rectum	12 (17.6)	11 (14.9)	23 (16.2)
**Time since diagnosis (months)**			
Mean (SD)	4.6 (3.0)	4.1 (3.2)	4.4 (3.2)
Range	1–12	1–12	1–12
**Initial treatment**			
Surgery	54 (79.4)	37 (50.0)	91 (64.1)
Chemotherapy/radiotherapy	9 (13.2)	10 (13.0)	19 (13.4)
Best Supportive care	5 (7.4)	27 (36.5)	32 (22.5)
**Anxiety score**			
0–7	27 (39.7)	48 (64.9)	75 (52.8)
8–21	41 (60.3)	26 (35.1)	67 (47.2)
**Depression score**			
0–7	26 (38.2)	35 (47.3)	61 (43.0)
8–21	42 (61.8)	39 (52.7)	81 (57.0)

### Anxiety and depression

The mean anxiety score was 7.6 (SD = 4.5) and for depression this was 8.7 (SD = 3.8). Overall 47.2% and 57% patients scored high on both anxiety and depression.

There were statistically significant differences between anxiety and depression and patients' knowledge of diagnosis. The results indicated that those who knew their diagnosis showed a significant higher degree of psychological distress [mean (SD) anxiety score: knew diagnosis 9.1 (4.2) vs. 6.3 (4.4) did not know diagnosis, P < 0.001; mean (SD) depression score: knew diagnosis 9.1 (4.1) vs. 7.9 (3.6) did not know diagnosis, P = 0.05]. Considering patients' demographic status, the findings showed different features indicating that there were no statistically significant differences between anxiety, depression and gender, patients' educational level, marital status, and cancer site. However, age and anxiety showed a significant relationship (P = 0.005) indicating that patients aged between 30 to 39 were more anxious compared with others. The results are shown in Table 2.

**Table 2:** Anxiety and depression scores for patients with gastrointestinal cancer by age, gender, marital status, educational status, cancer site and knowledge of diagnosis

	**Anxiety**	**Depression**
**Age**	**Mean (SD)**	**Mean (SD)**

19–29	8.4 (4.6)	8.0 (4.3)
30–39	10.6 (5.1)	8.9 (3.7)
40–49	9.1 (3.9)	9.4 (4.3)
50–59	7.8 (3.9)	8.5 (4.0)
≥60	6.2 (4.4)	8.1 (3.6)
*ANOVA*	*F = 3.87*	*F = 0.50*
*P value*	0.005*	*0.73*
**Gender**		
Male	7.0 (4.7)	8.4 (4.0)
Female	8.3 (4.2)	8.6 (3.7)
*t-test*	*-1.63*	*-0.021*
*P value*	*0.1*	*0.83*
**Marital status**		
Single	6.8 (2.7)	6.5 (2.9)
Married	7.7 (4.6)	8.6 (3.9)
Widowed	8.1 (5.1)	10.6 (3.1)
*ANOVA*	*F = 0.24*	*F = 2.75*
*P value*	*0.78*	*0.06*
**Educational status**		
Illiterate	7.2 (4.3)	8.6 (3.6)
Primary	7.2 (4.6)	7.6 (3.5)
Secondary	10.3 (4.9)	10.1 (5.9)
College/university	9.4 (3.2)	9.1 (3.4)
*ANOVA*	*F = 2.33*	*F = 1.64*
*P value*	*0.07*	*0.18*
**Cancer site**		
Esophagus	6.8 (4.5)	8.2 (3.5)
Stomach	8.0 (4.5)	8.9 (3.5)
Small intestine	5.0 (2.8)	8.2 (1.3)
Colon	8.4 (3.2)	7.8 (3.6)
Rectum	7.9 (5.8)	9.3 (5.4)
*ANOVA*	*F = 1.11*	*F = 0.67*
*P value*	*0.35*	*0.61*
**Initial treatment**		
Surgery	8.1 (4.4)	8.2 (3.9)
Chemotherapy/radiotherapy	6.6 (4.0)	8.7 (3.1)
Best Supportive care	6.9 (4.8)	8.9 (4.1)
*ANOVA*	*F = 1.30*	*F = 0.36*
*P value*	*0.27*	*0.69*
**Knowledge of diagnosis**		
Yes	9.1 (4.2)	9.1 (4.1)
No	6.3 (4.4)	7.9 (3.6)
*t-test*	*3.83*	*1.94*
*P value*	*<0.0001*	*0.05*

* Bonferroni correction indicated that only those in age group 30–39 and ≥60 significantly were differed.

Finally, performing regression analysis both anxiety and depression showed the strongest association with knowledge of diagnosis (odds ratio for anxiety: 2.7, 95% CI: 1.1–6.8, P = 0.03; odds ratio for depression: 2.8, 95% CI: 1.1–7.2, P = 0.03). No other variables studied showed significant results. The results are presented in Table 3.

**Table 3:** The results of logistic regression analysis

		**OR (95% CI)**	**P**
**Anxiety**			

	**Age**	0.97 (0.94–1.0)	0.11
	**Gender**		
	Male	1.0 (ref.)	
	Female	1.7 (0.78–3.7)	0.17
	**Marital status**		
	Married	1.0 (ref.)	
	Single/widowed	0.60 (0.16–2.1)	0.43
	**Educational Status**		
	Higher	1.0 (ref.)	
	Secondary	0.84 (0.10–7.5)	0.87
	Primary	0.22 (0.32–1.4)	0.11
	Illiterate	0.44 (0.06–3.2)	0.42
	**Cancer site**		
	Esophagus	1.0 (ref)	
	Stomach	1.4 (0.45–4.7)	0.52
	Small intestine	0.34 (0.03–4.2)	0.40
	Colon	0.80 (0.21–3.1)	0.75
	Rectum	0.62 (0.15–2.5)	0.51
	**Time since diagnosis**	1.1 (0.93–1.2)	0.34
	**Initial treatment**		
	Surgery	1.0 (ref.)	
	Chemotherapy/radiotherapy	0.35 (0.10–1.2)	0.11
	Best supportive care	1.3 (0.42–3.9)	0.64
	**Knowledge of cancer diagnosis**		
	No	1.0 (ref.)	
	Yes	2.7 (1.1–6.8)	0.03
**Depression**			
	**Age**	0.98 (0.95–1.0)	0.34
	**Gender**		
	Male	1.0 (ref.)	
	Female	1.3 (0.60–2.8)	0.50
	**Marital status**		
	Married	1.0 (ref.)	
	Single/widowed	.42 (0.12–1.4	0.16
	**Educational Status**		
	Higher	1.0 (ref.)	
	Secondary	0.26 (0.03–2.2)	0.22
	Primary	0.17 (0.02–1.2)	0.08
	Illiterate	0.73 (0.09–5.3)	0.73
	**Cancer site**		
	Esophagus	1.0 (ref.)	
	Stomach	1.9 (0.60–6.1)	0.27
	Small intestine	3.2 (0.34–29.8)	0.30
	Colon	0.74 (0.19–2.8)	0.66
	Rectum	0.95 (0.26–3.4)	0.94
	**Time since diagnosis**	1.0 (0.89–1.2)	0.78
	**Initial treatment**		
	Surgery	1.0 (ref.)	
	Chemotherapy/radiotherapy	2.2 (0.65–7.8)	0.19
	Best supportive care	1.5 (0.50–4.4)	0.47
	**Knowledge of cancer diagnosis**		
	No	1.0 (ref.)	
	Yes	2.8 (1.1–7.2)	0.03

OR = odds ratio, 95% CI = 95% confidence interval, ref. = reference group.

## Discussion

The main finding of the current study was the fact that we observed the lower levels of anxiety and depression in patients who did not know their cancer diagnosis. Similarly in Turkey and India it has been demonstrated that psychiatric disorders occur to lesser extent in patients who are not aware of their cancer diagnosis. The authors concluded that these patients had a more hopeful outlook to the outcome of treatment [23,24]. It is argued that since the majority of physicians in Iran do not inform caner patients about their true nature of illness, most patients who know their diagnosis obtain information indirectly, and thus this might lead to the higher level of emotional distress in patients who become aware of their illness [25,26]. However, a study of patients with advanced cancer suggested that awareness of prognosis does not itself cause depression [27].

The low level of knowledge of cancer diagnosis (48%) in this study was similar to those reported from Middle East countries [28,29]. One explanation for this finding could be due to the fact that in developing countries such as Iran the medical team cannot effectively address cancer patients' wishes and needs. The other main reason for not informing patients is that most people in Iran, as in many Middle East or Asian countries, interpret the diagnosis of cancer as equivalent to death and therefore patients' families may request physicians not to tell the patient the diagnosis and the word cancer [25,26]. A study in Nepal found that 63% of cancer patients were unaware of the nature of their disease while a survey of the general population showed that 80% of the respondents wanted to be informed if they were diagnosed with cancer [30]. Similarly, cancer patients in Taiwan expressed a strong preference for health care professionals to inform them of disease related information before disclosing information to their family members [31]. It has been suggested that the arguments that cancer patients from Asian cultures have different preferences regarding being informed of their cancer diagnosis and that family members have legitimate superior power in decision-making could not be supported from studies compiling data from these countries [32]. However, evidence suggests that sensible disclosure of diagnosis and prognosis is important and satisfaction with information-giving is associated with a better quality of life [33]. In addition, there seems to be a strong relationship between illiteracy and not knowing the diagnosis. The relevance of level of education and knowledge of cancer diagnosis seems worthwhile to be examined in the future studies. One might suggest that with regard to culture and resources of medical services in the Middle East there should be two different strategies for cancer disclosure: one for illiterate or less educated people and one for people with higher education.

There was no significant relationship between anxiety, depression and marital status, educational status and gender. A weaker association between demographic parameters and psychiatric morbidity has previously been noted in the presence of physical illness. It has been suggested that demographic differences that predispose to anxiety disorders after life events in the general population become less relevant when a very severe stressor such as cancer occurs [34].

There were no significant association between anxiety and depression and different gastrointestinal cancer site. In general when tumors develop in gastrointestinal tract, special problems in psychological adjustment are posed. Both patient and family may have to cope with severe eating problems, significant weight lost, nausea and vomiting, abdominal discomfort, diarrhea or constipation, as well as other disease related events that are difficult to manage. These problems result in immediate emotional distress and concerns [35]. Since the sample in this study was consisted of heterogeneous gastrointestinal cancer patients, common symptoms and discomfort in these people can be a possible reason for lack of relationship between anxiety, depression and different types of the cancer studied. In addition the number of some cancers in the current study were very small and thus to have a better understanding we recommend that for future studies to include patients from only one type of gastrointestinal tract.

A significant relationship was observed between anxiety and age. This is in accordance with previous studies demonstrating that young people are more distressed than elderly patients by serious illnesses such as cancer [13].

In general, because of the frightening and potentially stigmatizing nature of the cancer and high prevalence of psychological distress among cancer patients, physicians should be aware of how much difficulties the patients experience. They should detect and treat problems earlier or refer the patient to the psychiatrists more appropriately. Untreated psychiatric disorder in the presence of co-morbid conditions may result in more frequent clinic visits, increased costs, extended hospitalizations, and reduce compliance and quality of life [36].

## Conclusion

Overall 47.2% and 57% of patients with gastrointestinal cancer scored high on both anxiety and depression. Psychological distress was higher in those who knew their cancer diagnosis. It seems that the cultural issues and the way we provide information for cancer patients has an important role in their improved or decreased psychological status.

## Competing interests

The author(s) declare that they have no competing interests.

## Authors' contributions

AT was the main investigator and wrote the first draft of the manuscript. MAM contributed to patient recruitments. RR supervised the study. ZT contributed to the data collection. AM supervised the study, analyzed the data and wrote the final draft of the manuscript. SO contributed to the study design. All authors read and approved the final manuscript.

## Pre-publication history

The pre-publication history for this paper can be accessed here:


